# New types of topological superconductors under local magnetic symmetries

**DOI:** 10.1093/nsr/nwaa169

**Published:** 2020-07-24

**Authors:** Jinyu Zou, Qing Xie, Zhida Song, Gang Xu

**Affiliations:** Wuhan National High Magnetic Field Center & School of Physics, Huazhong University of Science and Technology, Wuhan 430074, China; Wuhan National High Magnetic Field Center & School of Physics, Huazhong University of Science and Technology, Wuhan 430074, China; Department of Physics, Princeton University, Princeton, NJ 08544, USA; Wuhan National High Magnetic Field Center & School of Physics, Huazhong University of Science and Technology, Wuhan 430074, China

**Keywords:** topological superconductor, magnetic symmetry, Majorana zero mode, superconducting wire

## Abstract

We classify gapped topological superconducting (TSC) phases of one-dimensional quantum wires with local magnetic symmetries, in which the time-reversal symmetry $\mathcal {T}$ is broken, but its combinations with certain crystalline symmetries, such as $M_x \mathcal {T}$, $C_{2z} \mathcal {T}$, $C_{4z}\mathcal {T}$ and $C_{6z}\mathcal {T}$, are preserved. Our results demonstrate that an equivalent BDI class TSC can be realized in the $M_x \mathcal {T}$ or $C_{2z} \mathcal {T}$ superconducting wire, which is characterized by a chiral *Z*^*c*^ invariant. More interestingly, we also find two types of totally new TSC phases in the $C_{4z}\mathcal {T}$ and $C_{6z}\mathcal {T}$ superinducting wires, which are beyond the known AZ class, and are characterized by a helical *Z*^*h*^ invariant and *Z*^*h*^⊕*Z*^*c*^ invariants, respectively. In the *Z*^*h*^ TSC phase, *Z* pairs of Majorana zero modes (MZMs) are protected at each end. In the $C_{6z}\mathcal {T}$ case, the MZMs can be either chiral or helical, and even helical-chiral coexisting. The minimal models preserving $C_{4z}\mathcal {T}$ or $C_{6z}\mathcal {T}$ symmetry are presented to illustrate their novel TSC properties and MZMs.

## Teaser

Topological superconducting phases of one-dimensional quantum wires with local magnetic symmetries are classified and new types of Majorana modes are discussed.

## INTRODUCTION

Topological superconductors (TSCs) are new kinds of topological quantum states, which are fully superconducting gapped in the bulk but support gapless excitations called Majorana zero modes (MZMs) at the boundaries [1–5]. As analogues of the famous Majorana fermions [6], MZMs are their own antiparticles, and are proposed as the qubits of topological quantum computation because of their nonlocal correlation and non-Abelian statistic nature [7–10]. Hence, searching for TSC materials with MZMs is now an important topic in condensed matter physics, and a series of schemes have been proposed in the last decade, including the proximity effect on the surface of topological insulators [11–16] and the recently predicted intrinsic superconducting topological materials [17–25].

To identify whether a superconductor is topologically nontrivial, we should first ascertain to what topological classification it belongs. The topological classification can be highly enriched by symmetries, including time-reversal symmetry $\mathcal {T}$, particle-hole symmetry $\mathcal {P}$ and especially the crystalline symmetries [26–35]. The topology for noninteracting Hamiltonians of the 10 Altland–Zirnbauer (AZ) classes with or without $\mathcal {T}$ and $\mathcal {P}$ has been well classified [26,27]. Particularly, the Bogoliubov–de Gennes (BdG) Hamiltonians of the one-dimensional (1D) superconductors, with $\mathcal {T}$ breaking or preserving, belong to the D and DIII classes, respectively. In both cases we only have the *Z*_2_ classification. In addition to these local symmetries, crystalline symmetries are considered for each AZ class to generalize the topological classification [28–31], and the topological crystalline superconductors protected by mirror reflection symmetry [32,33] or rotational symmetries  [34,35] have been proposed. Furthermore, the TSC phase protected by the magnetic symmetries $M_x\mathcal {T}$ and $C_{2z}\mathcal {T}$ has been discussed in [34,36–38]. Nevertheless, the topological classification of superconductors with general magnetic symmetries is still an open question, and the corresponding theoretical analysis is necessary for understanding and searching for new magnetic TSC materials and MZMs.

In this paper, we focus on the topological phases of gapped superconducting wires with local magnetic symmetries (LMSs), in which $\mathcal {T}$ is broken, but its combinations with certain crystalline symmetries—those leaving each site invariant, including $M_x \mathcal {T}$, $C_{2z} \mathcal {T}$, $C_{4z}\mathcal {T}$ and $C_{6z}\mathcal {T}$—are preserved. Our analysis shows that, with $M_x\mathcal {T}$ or $C_{2z}\mathcal {T}$ symmetry, an effective BDI class TSC can be realized, which is characterized by a chiral *Z*^*c*^ topological invariant and protects an integer number of MZMs at each end. Remarkably, two totally new TSC phases are discussed in the superconducting wire with $C_{4z}\mathcal {T}$ or $C_{6z}\mathcal {T}$ symmetry. In the $C_{4z}\mathcal {T}$ case, the BdG Hamiltonian is characterized by a helical *Z*^*h*^ invariant, which can protect *Z* pairs of MZMs at each end. The BdG Hamiltonian with $C_{6z}\mathcal {T}$ symmetry possesses *Z*^*h*^⊕*Z*^*c*^ invariants, which means that the helical and chiral MZMs can coexist in a single wire system. The minimal models with the LMSs $C_{4z}\mathcal {T}$ and $C_{6z}\mathcal {T}$ are presented separately, in which the TSC with helical MZMs and the TSC with helical-chiral coexisting MZMs are discussed. Our results may facilitate the ongoing search for novel TSCs.

## TOPOLOGICAL CLASSIFICATION OF GAPPED SUPERCONDUCTING WIRE

We first introduce the LMSs for a magnetic superconducting wire along the $z$ direction. Among the 1D space groups (the so-called rod group) [39], the local symmetry operators include the mirror reflection *M*_*x*_ and the *n*-fold rotation *C*_*nz*_ with *n* = 2, 3, 4, 6. Combined with $\mathcal {T}$, we obtain four types of LMSs, $\mathcal {T}^\prime = M_x\mathcal {T}$, $C_{2z}\mathcal {T}$, $C_{4z}\mathcal {T}$ and $C_{6z}\mathcal {T}$, as tabulated in Table 1. We consider a 1D BdG Hamiltonian preserving $\mathcal {T}^\prime$. Note that the operation of $\mathcal {T}^\prime$ does not change the positions of electrons. Hence, it acts on the BdG Hamiltonian like a time-reversal operator

(1) \begin{eqnarray*} \mathcal {T}^\prime H_{\text{BdG}}(k) \mathcal {T}^{\prime -1} = H_{\text{BdG}}(-k). \end{eqnarray*}

Here, LMS $\mathcal {T}^\prime$ takes the form $\mathcal {T}^\prime =U\mathcal {K}$ with $\mathcal {K}$ being the complex conjugate operator and *U* being a unitary matrix determined by the spatial operation and spin flipping. We employ the convention that $[\mathcal {T},\mathcal {P}] = 0$ and set $\mathcal {P} = \tau _x \mathcal {K}$, where the Pauli matrix τ_*x*_ acts on the particle-hole degree of freedom. Combining $\mathcal {T}^\prime$ and $\mathcal {P}$ leads to a chiral symmetry $\mathcal {S}=\mathcal {T}^\prime \mathcal {P}$. Both $\mathcal {P}$ and $\mathcal {S}$ act on the BdG Hamiltonian as

(2) \begin{eqnarray*} \mathcal {P}H_{\text{BdG}}(k)\mathcal {P}^{-1} = -H_{\text{BdG}}(-k), \end{eqnarray*}

(3) \begin{eqnarray*} \mathcal {S}H_{\text{BdG}}(k)\mathcal {S}^{-1} = -H_{\text{BdG}}(k). \end{eqnarray*}

**Table 1.** The topological classification of the 1D gapped superconducting systems with the LMSs $M_x\mathcal {T}$, $C_{2z}\mathcal {T}$, $C_{4z}\mathcal {T}$ and $C_{6z}\mathcal {T}$, respectively. 2×AIII form a helical *Z*^*h*^ classification.

$\mathcal {T}^\prime$	$M_x \mathcal {T}$	$C_{2z}\mathcal {T}$	$C_{4z}\mathcal {T}$	$C_{6z}\mathcal {T}$
	(*n* = 2)	(*n* = 2)	(*n* = 4)	(*n* = 6)
$\mathcal {T}^{\prime n}$	1	1	−1	1
$\mathcal {P}^2$	1	1	1	1
$\mathcal {S}^n$	1	1	−1	1
Invariant	*Z* ^*c*^	*Z* ^*c*^	*Z* ^*h*^	*Z* ^*h*^⊕*Z*^*c*^
	(BDI)	(BDI)	(2×AIII)	(2×AIII ⊕ BDI)

The chiral symmetry $\mathcal {S}$ has a series of eigenvalue pairs ±*s*_1_, ±*s*_2_, … and it can take a block-diagonal form as $\mathcal {S} =\text{diag}[\mathcal {S}_{\pm {s_1}}, \mathcal {S}_{\pm {s_2}}, \ldots ]$, where the subscript ±*s*_1_ denotes the direct sum of eigenvector spaces |*s*_1_〉 and | − *s*_1_〉. The anticommute relation (3) means that *H*_BdG_(*k*) can be block diagonalized according to the eigenvalues of $\mathcal {S}^2$. In other words, *H*_BdG_(*k*) can adopt the form $H_{\text{BdG}}(k) = \text{diag} [ H_{s_1^2}, H_{s_2^2}, \ldots ]$. Hence, the topological classification of the whole Hamiltonian is decomposed into examining the topology of each block and their compatibility. For each block Hamiltonian $H_{s^2}$, its topology is equivalent to either the BDI or the AIII class, depending on the chiral symmetry eigenvalue *s*. To be specific, when *s* is a real number, $H_{s^2}$ is invariant under $\mathcal {T}^{\prime }$ or $\mathcal {P}$, which means that it belongs to the BDI class and possesses a *Z* invariant expressed as $v$ = *N*_*s*_ − *N*_−*s*_, where the *N*_±*s*_ are the numbers of MZMs with chiral symmetry eigenvalue ±*s*, respectively. Additionally, when *s* is a complex number, $H_{s^2}$ is transformed into $H_{s^{\ast 2}}$ under $\mathcal {T}^\prime$ or $\mathcal {P}$. Hence, the $H_{s^2}$ ($H_{s^{\ast 2}}$) belongs to the AIII class that is characterized by a *Z* invariant $v$ = *N*_*s*_ − *N*_−*s*_ ($v= N_{s^\ast } - N_{-s^\ast }$), which is equal to the number of MZM pairs on each wire end.

We next consider the compatibility between the different MZMs possessing different $\mathcal {S}$ eigenvalues. To do this, we introduce a coupling term *m*|*s*_1_〉〈*s*_2_|, which satisfies the chiral symmetry, i.e. $\mathcal {S} m|s_1\rangle \langle s_2 | \mathcal {S}^{-1} = - m|s_1\rangle \langle s_2 | = ms_1 s_2^\ast |s_1\rangle \langle s_2 |$. Here *m* is a perturbation parameter, and |*s*_1_〉 and |*s*_2_〉 are the eigenstates of $\mathcal {S}$. Then we see that *m* can be nonzero only when $s_1 s_2^\ast = -1$, which means that MZMs within one block having chiral eigenvalues *s* and −*s* can couple to each other and be eliminated. However, MZMs from different blocks are noninterfering due to the protection of $\mathcal {S}$. Therefore, the topological classification of the whole BdG Hamiltonian is determined by the summation of the topology for each block. We summarize the topological classification of 1D gapped superconductors in Table 1 and analyse each case in the following.

**Figure 1.**
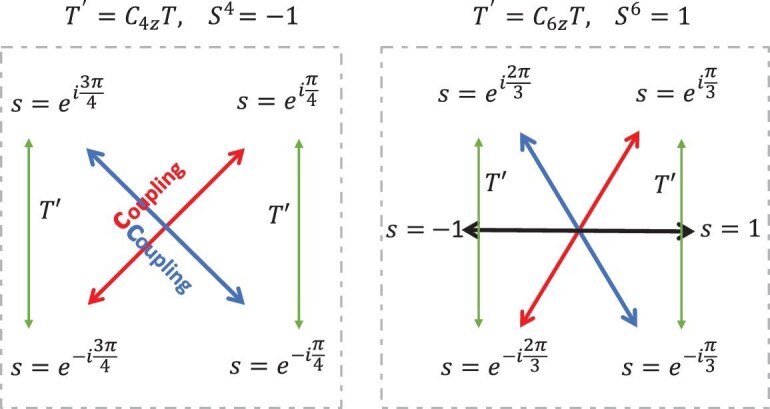
The eigenvalues of $\mathcal {S}$ and their transformations in the $C_{4z}\mathcal {T}$ and $C_{6z}\mathcal {T}$ cases. Complex conjugating partners *s* and s^*^ are related by the LMSs and always coexist. A perturbation term can be introduced to couple the chiral states with opposite eigenvalues, as illustrated by the red, blue and black double-head arrows.

- *
$M_x\mathcal {T}$ and $C_{2z}\mathcal {T}$ cases*. These two cases are equivalent to the BDI class with $\mathcal {T}^{\prime 2}=1$ and $\mathcal {S}^2=1$. The chiral topological invariant $v$ = *N*_1_ − *N*_−1_ ∈ *Z* is given by the winding number [5,26]
  (4) \begin{eqnarray*} v = \frac{1}{2\pi }\int dk \text{Tr}[W^\dagger (k)\partial _k W(k)]\\ = \frac{1}{2\pi } \int dk \partial _k \theta (k). \end{eqnarray*}
  Here *W*(*k*) is a unitary matrix that diagonalizes the BdG Hamiltonian and θ(*k*) is the phase angle of Det[*W*(*k*)]. The identity Tr[ln(*W*)] = ln(Det[*W*]) is used to derive the above equation. These results agree well with the conclusions reached in previous studies [34,36–38,40,41].
- *
$C_{4z}\mathcal {T}$ case*. The chiral symmetry satisfies $\mathcal {S}^4=-1$ and has eigenvalues ±*e*^±*i*π/4^ (see Fig. 1). We can conclude that the topological invariants are given by $v = N_{e^{i\pi /4}} - N_{-e^{i\pi /4}}$ (or $N_{e^{-i\pi /4}} - N_{-e^{-i\pi /4}})$. The TSC phase is hence characterized by the helical topological invariant $v$ ∈ *Z*, which means that the MZMs always appear in Kramers pairs. This is obviously different from the chiral *Z* invariant in the BDI class, in which the MZMs can arise one by one as *Z* increases. To distinguish the chiral *Z* and helical *Z* invariants, we use *Z*^*c*^ and *Z*^*h*^ in the following. The *Z*^*h*^ TSC phase of the $C_{4z}\mathcal {T}$-preserving wire can be understood from the following perspective. The BdG Hamiltonian can be block diagonalized into two sectors according to the eigenvalues ±*i* of $C_{2z}=(C_{4z}\mathcal {T})^2$ as *H*_*i*_(*k*)⊕*H*_−*i*_(*k*). Both $C_{4z}\mathcal {T}$ and $\mathcal {P}$ can map these two sectors to each other. However, their combination, i.e. the chiral symmetry $\mathcal {S}$, keeps each sector invariant. As a consequence, each sector belongs to the AIII class, whose *Z*^*c*^ topological invariant can be calculated by exploiting (4). Yielding to the $C_{4z}\mathcal {T}$ symmetry, the *Z*^*c*^ invariants of two sectors must be equal, which finally gives a *Z*^*h*^ invariant for the whole BdG Hamiltonian. That is, the topological invariant $v$ is given by the winding number of each *C*_2$z$_ eigenvalue sector as defined in (4).
- *
$C_{6z}\mathcal {T}$ case*. We have $\mathcal {T}^{\prime 6}=1$ and $\mathcal {S}^6=1$. As illustrated in Fig. 1, the chiral symmetry has eigenvalues ±*e*^±*i*π/3^, ±1. The topology is characterized by *Z*^*h*^⊕*Z*^*c*^ invariants that are given by $N_{e^{\pm i\pi /3}} -N_{-e^{\pm i\pi /3}}$ and *N*_1_ − *N*_−1_, respectively. Similar to the $C_{4z}\mathcal {T}$ case, the BdG Hamiltonian can be block diagonalized as $H= H_{e^{i2\pi /3}} \oplus H_{e^{-i2\pi /3}} \oplus H_{1}$ according to the eigenvalues *e*^±*i*2π/3^, 1 of $C_{3z}=(C_{6z}\mathcal {T})^2$. The $H_{e^{i2\pi /3}}$ and $H_{e^{-i2\pi /3}}$ sectors both belong to the AIII class, forming a *Z*^*h*^ classification together, whereas the *H*_1_ sector itself forms a *Z*^*c*^ classification (i.e. BDI class) with $\mathcal {P}$ and an effective $\mathcal {T}_{\text{eff}} = (C_{6z}\mathcal {T})^3$. Therefore, the topology of the whole BdG Hamiltonian is classified by *Z*^*h*^⊕*Z*^*c*^, whose topological invariants ($v$^*h*^, $v$^*c*^) are given by the winding numbers of the $H_{e^{i2\pi /3}}$ and *H*_1_ sectors, respectively. As a consequence, in a 1D superconducting wire with the LMS $C_{6z}\mathcal {T}$, the helical and chiral MZMs can coexist. Such novel TSC phase stimulate further interests in the manipulation of such helical-chiral coexisting MZMs [42–44].

## MODEL REALIZATION

**Figure 2.**
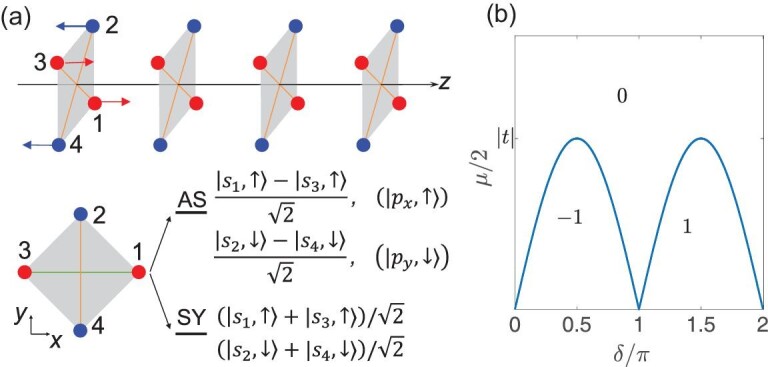
(a) A $C_{4z}\mathcal {T}$-preserving superconducting wire aligned along the $z$ direction, in which the red and blue dots denote the spin up (+$z$) and spin down (−$z$) polarized *s* orbitals, respectively. The intra-cell coupling between the same spin orbitals is much larger than the spin-orbit coupling, which split the four states into one symmetric (SY) manifold and one antisymmetric (AS) manifold. Both manifolds are double degenerate. For simplicity, only the AS manifold is considered in our tight-binding model (5). (b) The topological phase diagram of (8) as the function of μ and δ, in which 0, ±1 are the winding numbers, μ is the chemical potential and δ = π/2 + φ − α is the phase difference between the coefficients of τ_*y*_ and τ_$z$_.

To illustrate the TSC phase with the LMS $C_{4z}\mathcal {T}$, we construct a 1D antiferromagnetic chain along the $z$ direction, as shown in Fig. 2(a), where each unit cell contains four subsites and each subsite is occupied by one spin polarized *s* orbital. We consider that the intra-cell coupling between the same spin states is much larger than the spin-orbit coupling, and thus the four orbitals are well split into two double-degenerate manifolds, as illustrated in Fig. 2(a). More details of the full model have been given in the online supplementary material. Here, to capture the topological phase of the model, we take the |*p*_*x*_, ↑〉 and |*p*_*y*_, ↓〉 subspaces to build an effective tight-binding model. Up to the nearest-neighbor hopping, it can be written as

(5) \begin{eqnarray*} H_{\text{TB}}^{\rm eff} &=& \sum _l t c^{\dagger}_{l+1,p_x,\uparrow } c^{}_{l,p_x,\uparrow } + t^\ast c^\dagger_{l+1,p_y,\downarrow } c^{}_{l,p_y,\downarrow } \\ +\,\, h.c.+ \mu \sum _{l,\sigma } c^\dagger_{l\sigma } c^{}_{l\sigma }, \end{eqnarray*}

where *t* = |*t*|*e*^*i*α^ is the complex hopping, μ is the chemical potential and σ acts on the orbital degree of freedom of the |*p*_*x*_, ↑〉 and |*p*_*y*_, ↓〉 states. The $C_{4z}\mathcal {T}$ is given by $e^{i\pi /4\sigma _z}\sigma _y\mathcal {K}$. Note that the hopping terms between opposite spins are prohibited by the *C*_2$z$_ symmetry. The *s*-wave pairing Hamiltonian takes the form

(6) \begin{eqnarray*} H_{\Delta } &=& \sum _l \Delta c^{\dagger}_{l+1,p_x,\uparrow } c^{\dagger}_{l,p_y,\downarrow } + \Delta ^\ast c^\dagger_{l+1,p_y,\downarrow } c^{\dagger}_{l,p_x,\uparrow }\\ +\,\, h.c., \end{eqnarray*}

with Δ = |Δ|*e*^*i*φ^. The pairing terms between the same spin are also prohibited by the *C*_2$z$_ symmetry.

In the Nambu basis $(c_{k,p_x,\uparrow }, c^\dagger_{-k,p_y,\downarrow }, c_{k,p_y,\downarrow }, c^\dagger_{-k,p_x,\uparrow } )^T$, $\mathcal {P}$ and $\mathcal {T}^\prime$ are given by $\mathcal {P} = \sigma _x \otimes \tau _x \mathcal {K}$ and $\mathcal {T}^\prime = e^{i\pi /4 \sigma _z} \sigma _y \otimes I \mathcal {K}$, respectively, which give $\mathcal {S} = e^{-i\pi /4 \sigma _z} \otimes \tau _x$. The BdG Hamiltonian anticommutes with $\mathcal {S}$ and takes a block-diagonal form as

(7) \begin{eqnarray*} H_{\text{BdG}}^{C_{4z}\mathcal {T}}(k) = \left({\begin{array}{cc}H_{i}(k) &\quad \\ &\quad H_{-i}(k) \\ \end{array}}\right) \end{eqnarray*}

with

(8) \begin{eqnarray*} H_{\pm i}(k) &=& |t| \cos (k\pm \alpha ) \tau _z - |\Delta | \sin (k\pm \phi ) \tau _y \\ +\, \frac{\mu }{2} \tau _z . \end{eqnarray*}

Then the spectrum is given by

(9) \begin{eqnarray*} E(k) =\\ \pm \sqrt{ \left[|t| \cos (k\pm \alpha ) +\frac{\mu }{2}\right]^2 + |\Delta |^2 \sin ^2(k\pm \phi ) }. \end{eqnarray*}

Note that the two blocks in (7) are Kramers pairs related by the $C_{4z}\mathcal {T}$ symmetry and have the same winding number. A straightforward way to determine the topology is to calculate the winding number $v$ using (4) for the upper or lower blocks. Here we provide a much simpler way to obtain $v$ by analogizing the coefficients of the block Hamiltonians with elliptically polarized lights, whose electric field is described by *E*_*x*_ = *A*_*x*_cos (*kz* − ω*t*), *E*_*y*_ = *A*_*y*_cos (*kz* − ω*t* + δ). In the following we analyze the winding number of *H*_*i*_(*k*), where the coefficients of the Pauli matrices are *h*_$z$_ − μ/2 = |*t*|cos (*k* + α) and *h*_*y*_ = |Δ|sin (*k* + φ). When |*t*||sin δ| > μ/2 (<μ/2), the parameter curve of *h*_*y*_(*k*) and *h*_$z$_(*k*) will (not) wind around the zero point *h*_$z$_ = *h*_*y*_ = 0 (we assume that μ > 0 for simplicity), and the superconducting wire is in a topological nontrival (trivial) phase. Furthermore, when δ ∈ (0, π) [δ ∈ (−π, 0)], we have a left-handed (right-handed) parameter curve, and the topological phase is characterized by winding number +1 ( − 1). The phase diagram in the δ − μ parameter space is plotted in Fig. 2(b). We point out that, when next-nearest-neighbor hopping and pairing are considered, the competition with nearest-neighbor hopping and pairing gives rise to the opportunity for TSC phase with higher winding numbers.

**Figure 3.**
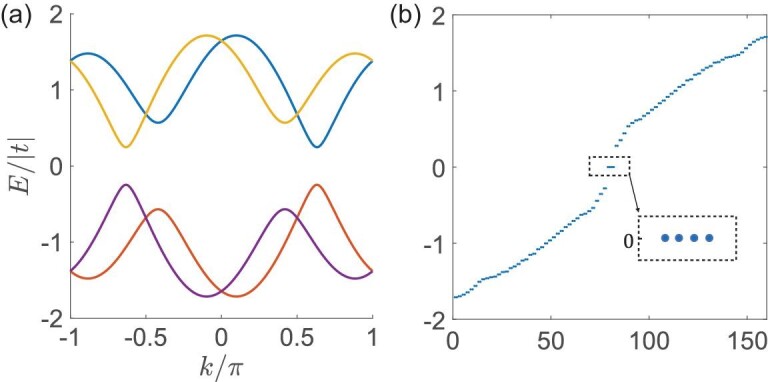
The bulk spectrum and MZMs in the $C_{4z}\mathcal {T}$-preserving TSC model. (a) The gapped bulk spectrum of the $C_{4z}\mathcal {T}$-preserving TSC phase with *t* = 1, Δ = 1.3*e*^*i*π/3^, μ = 0.2. (b) The corresponding spectrum of (a) with an open boundary on both sides, in which four MZMs appear at zero energy.

In the nontrivial TSC phase, the open quantum wire traps integer pairs of MZMs at its ends. By using *t* = 1, Δ = 1.3*e*^*i*π/3^, μ = 0.2, we observe two pairs of MZMs in total on the open wire spectrum, as shown in Fig. 3(b), which is in contrast with the gapped bulk spectrum in Fig. 3(a). These MZMs can also be solved from the continuous low-energy model [45]. Here, we need only consider *H*_*i*_(*k*) since the other block in (7), as well as its zero energy solution, can be obtained by a $C_{4z}\mathcal {T}$ transformation. By assuming that the wire is placed on the $z$ > 0 side, the low-energy massive Dirac Hamiltonian close to *k* = π/2 is given by

(10) \begin{eqnarray*} \!\!\!\!H_{i} =\\ \!\!\!\!\left({\begin{array}{cc}-i|t|\partial _z + \mu /2 &\,\, |\Delta | (-i \sin \delta \!+\! \cos \delta \partial _z ) \\ |\Delta | ( i\sin \delta \!-\! \cos \delta \partial _z) &\,\, i |t| \partial _z - \mu /2 \\ \end{array}}\right). \end{eqnarray*}

Its zero energy solution Ψ_1_ and the $C_{4z}\mathcal {T}$-related partner $\Psi _2 = C_{4z}\mathcal {T}\Psi _1$ are given by

(11) \begin{eqnarray*} \Psi _1= \left({\begin{array}{c}1 \\ 1 \\ 0 \\ 0 \\ \end{array}}\right) \exp \bigg [{\int dz \frac{i|\Delta | \sin \delta -\mu /2 }{ |\Delta | \cos \delta - i |t| }}\bigg ] , \end{eqnarray*}

(12) \begin{eqnarray*} \Psi _2= \left({\begin{array}{c}0 \\ 0 \\ 1 \\ 1 \\ \end{array}}\right) \exp \bigg [{\int dz \frac{ - i|\Delta | \sin \delta -\mu /2}{ |\Delta | \cos \delta + i |t| }} \bigg ] . \end{eqnarray*}

These two states are the eigenstates of the chiral symmetry $\mathcal {S}$ with eigenvalues *e*^±*i*π/4^, respectively. Therefore, they are immune to perturbations preserving $\mathcal {S}$ (and $C_{4z}\mathcal {T}$). Their combinations give the $\mathcal {S}$ protected MZMs as γ_1_ = Ψ_1_ + Ψ_2_ and γ_2_ = *i*(Ψ_1_ − Ψ_2_).

The $C_{4z}\mathcal {T}$-preserving BdG Hamiltonian can be easily generalized to a $C_{6z}\mathcal {T}$ invariant quantum wire. For this purpose, we assume that the chiral symmetry is expressed as $\mathcal {S}=(e^{-i\pi /3\tau _z} \otimes \tau _x) \oplus \tau _x$. The BdG Hamiltonian can then be written in three blocks $H_{\text{BdG}}^{C_{6z}\mathcal {T}} = H_{e^{i2\pi /3}}(k) \oplus H_{e^{-i2\pi /3}}(k) \oplus H_{1}(k)$ with

(13) \begin{eqnarray*} H_{e^{\pm i2\pi /3}}(k) & =& |t| \cos (k\pm \alpha )\tau _z \\ -\,\, |\Delta | \sin (k\pm \phi ) \tau _y + \frac{\mu }{2} \tau _z, \\ H_{1}(k) & =& |t^{\prime }|\cos (k)\tau _z -|\Delta ^{\prime }|\sin (k)\tau _y\\ +\,\, \frac{\mu }{2}\tau _z. \end{eqnarray*}

The first two blocks are Kramers pairs and take the same form as in (8), while the last block is transformed to itself under $C_{6z}\mathcal {T}$ or $\mathcal {P}$. For this BdG Hamiltonian, the topology is characterized by *Z*^*h*^⊕*Z*^*c*^ numbers, which correspond to the number of helical and chiral MZMs, respectively. The topological phase diagram of the helical part Hamiltonian is the same as in Fig. 2(b). The chiral part is determined by the winding number of *H*_1_(*k*), which gives a nontrivial TSC phase when |*t*′| > μ/2.

## CONCLUSION

We have classified the TSC phases of quantum wires with LMSs. In the case of $M_x\mathcal {T}$ or $C_{2z}\mathcal {T}$, an equivalent BDI class TSC can be realized [37,38,40]. More importantly, we find two new types of TSC phases in the superconducting wire with $C_{4z}\mathcal {T}$ or $C_{6z}\mathcal {T}$, which are beyond the already known AZ classes and can be characterized by *Z*^*h*^ or *Z*^*h*^⊕*Z*^*c*^ topological invariants, respectively. These results not only enrich the variety of the 1D TSC, but also provide luxuriant building blocks for the construction of new type 2D and 3D TSCs, by following the general method proposed in [46]. For example, one can couple the 1D TSCs in the *y* direction to construct a 2D TSC. The high symmetry lines *k*_*y*_ = 0 and *k*_*y*_ = π in momentum space preserve the 1D LMS. With proper parameters, the *k*_*y*_ = 0 and *k*_*y*_ = π lines can belong to distinct topological phases, and result in the gapless propagating Majorana edge states connecting the conducting bands and valence bands. The superconductivity and antiferromagnetism coexisting SmOFeAs [47,48] with a proper magnetic configuration satisfying *C*_4$z$_*T* symmetry is a possible material to study the 1D TSC phase on its high-symmetry lines.

## Supplementary Material

nwaa169_Supplemental_File
