# Supplementary material for: New types of topological superconductors under local magnetic symmetries
Source: Natl Sci Rev. 2020 Jul 24;8(5):nwaa169. doi: 10.1093/nsr/nwaa169 (PMC8288464; doi:10.1093/nsr/nwaa169)
Supplement: nwaa169_Supplemental_File [file nwaa169_supplemental_file.pdf]

Supplementary material

In this Supplementary material, we give the detail Hamiltonian of the full model in Fig.2(a), and numerically calculate the Majorana zero modes, which are consistent with the results of the effective model. The tight-binding Hamiltonian is given as

$$\begin{aligned}H_{TB} = &\sum_l m(c^\dagger_{l,3\uparrow}c_{l,1\uparrow} + c^\dagger_{l,4\downarrow}c_{l,2\downarrow}) + \lambda(c^\dagger_{l,1\uparrow}c_{l,2\downarrow} - c^\dagger_{l,3\uparrow}c_{l,4\downarrow}) - i\lambda^*(c^\dagger_{l,2\downarrow}c_{l,3\uparrow} - c^\dagger_{l,4\downarrow}c_{l,1\uparrow}) \\ &- t(c^\dagger_{l+1,3\uparrow}c_{l,1\uparrow} + c^\dagger_{l+1,1\uparrow}c_{l,3\uparrow}) - t^*(c^\dagger_{l+1,4\downarrow}c_{l,2\downarrow} + c^\dagger_{l+1,2\downarrow}c_{l,4\downarrow}) + h.c. \\ &+ \sum_{l,\sigma}\mu' c^\dagger_{l,\sigma}c_{l,\sigma},\end{aligned} \tag{1}$$

where the binding energy m and spin-orbit coupling λ separate the symmetry manifold and antisymmetry manifold as shown in Fig.2(a). The splitting plus the chemical potential μ' gives the effective chemical potential μ in Eq.(5). The paring Hamiltonian takes the form as

$$\begin{aligned}H_{pair} = &\sum_l \Delta c^\dagger_{l+1,1\uparrow}c^\dagger_{l,2\downarrow} - \Delta^* c^\dagger_{l+1,2\downarrow}c^\dagger_{l,3\uparrow} + \Delta c^\dagger_{l+1,3\uparrow}c^\dagger_{l,4\downarrow} - \Delta^* c^\dagger_{l+1,4\downarrow}c^\dagger_{l,1\uparrow} \\ &+ \sum_l -\Delta^* c^\dagger_{l,1\uparrow}c^\dagger_{l+1,2\downarrow} + \Delta c^\dagger_{l,2\downarrow}c^\dagger_{l+1,3\uparrow} - \Delta^* c^\dagger_{l,3\uparrow}c^\dagger_{l+1,4\downarrow} + \Delta c^\dagger_{l,4\downarrow}c^\dagger_{l+1,1\uparrow} \\ &+ h.c.\end{aligned} \tag{2}$$

The numerical calculation confirms two pairs of Majorana zero modes on the open wire spectrum, as shown in Fig. S1.

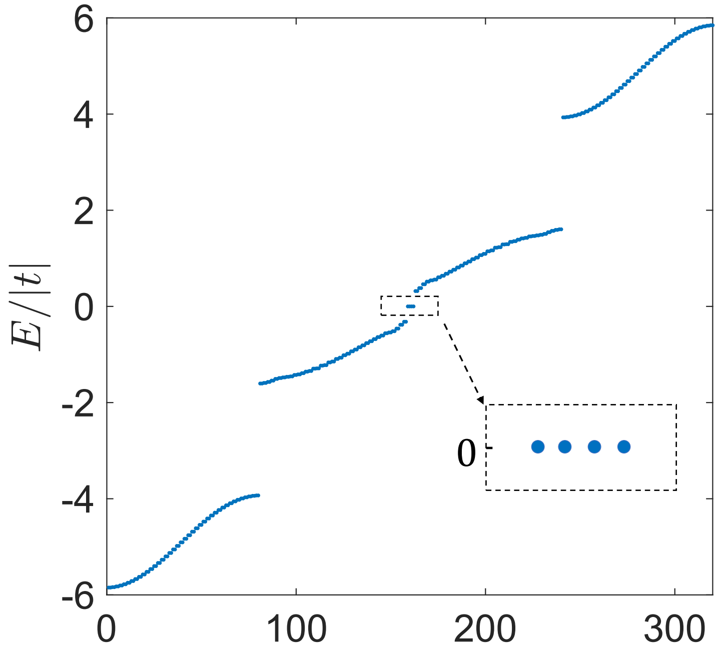

FIG. 1. The energy spectrum and MZMs of the $C_{4z}\mathcal{T}$-preserving full model with an open boundary on both sides, the parameters are $t = 1, \Delta = 1.3e^{i\pi/3}, m = -2.3, \lambda = 0.4, \mu' = -2.5$.
